# Future MS care: a consensus statement of the MS in the 21st Century Steering Group

**DOI:** 10.1007/s00415-012-6656-6

**Published:** 2012-08-31

**Authors:** Peter Rieckmann, Alexey Boyko, Diego Centonze, Alasdair Coles, Irina Elovaara, Eva Havrdová, Otto Hommes, Jacques LeLorier, Sarah A. Morrow, Celia Oreja-Guevara, Nick Rijke, Sven Schippling

**Affiliations:** 1 Department of Neurology, Sozialstiftung Bamberg Hospital, Bamberg, Germany; 2 Moscow MS Center, Dvinstev 6, City Hospital 11, Moscow, Russia; 3 Clinical and Research MS Center, Department of Neuroscience, Tor Vergata University, Viale Oxford 81, 00133 Rome, Italy; 4 Department of Neurology, Box 165, Addenbrooke’s Hospital, Hills Road, Cambridge, CB2 0QQ UK; 5 Department of Neurology, Tampere University Hospital and University of Tampere, Medical School, A Building, 33014 Tampere, Finland; 6 Department of Neurology, First Medical Faculty, Charles University, Kateřinská 30, 128 08 Prague 2, Czech Republic; 7 European Charcot Foundation, Hoeveveld 18a, 6584 GG Molenhoek (Nijmegen area), The Netherlands; 8 Pavillon Masson, 3850 Saint-Urbain Street, Montreal, QC H2W 1TF Canada; 9 Department of Neurology, Western University, LHSC-UH, B10-118, 339 Windermere Rd, London, ON N6A 5A5 Canada; 10 Department of Neurology, University Hospital San Carlos, Prof. Martin Lagos s/n, 28040 Madrid, Spain; 11 MS National Centre, 372 Edgeware Road, London, NW2 6ND UK; 12 Department of Neuroimmunology and MS Research (NIMS), Neurology Clinic, University Medical Center Zurich, Frauenklinikstrasse 26, 8091 Zurich, Switzerland

**Keywords:** Multiple sclerosis, Care, Management, Consensus statement

## Abstract

The “MS in the 21st Century” initiative was established with the purpose of (1) defining how multiple sclerosis (MS) treatment and standards of care should look in the 21st century; (2) developing a minimum standard of care across the world; and (3) motivating the broad MS community to align standards of care and challenge the current treatment paradigm. The aim was to develop a consensus statement to reach and influence the broader MS community. An expert steering group from Europe and Canada—consisting of neurologists, patient advocates, a pharmacoepidemiologist/pharmacoeconomist, and representatives from national MS centers—participated in a series of workshop-driven meetings between February 2011 and 2012. After three phases of discussions, the steering group identified that the overall vision for future care of MS should be full access to personalized treatment, with reimbursement, to achieve freedom from disease. They constructed seven overall principles that support this vision: personalized care, patient engagement, commitment to research, regulatory body education and reimbursement issues, new endpoints in clinical trials, more therapy options, and MS centers of excellence. This consensus statement outlines the key aspects of the seven principles that need to be addressed. The “MS in the 21st Century Steering Group” hopes that this consensus statement acts as a call to action for healthcare providers and decision-makers to address simultaneously the overarching principles that will guide patient management in order to improve outcomes for people with MS.

## Introduction

More than 2.5 million people worldwide are estimated to be affected by multiple sclerosis (MS), with almost 500,000 of them living in the European Union (EU) [1]. Current medications slow the accumulation of disability in MS and reduce the frequency of relapses by modulating or (selectively) suppressing the patient’s immune system [2]. Although scientific evidence exists to show that early access to effective treatment and care is cost-efficient to society and has a meaningful impact on the quality of life of patients [3], significant disparity still exists between and within countries in the standard of treatment offered to people with MS [4]. There also remains a lack of awareness and education of MS in general among healthcare professionals, patients, and the general public [5–8]. In addition, available treatments have limited efficacy [9].

In February 2011, the MS in the 21st Century initiative was established with the purpose of (1) defining how MS treatment and standards of care should look in the 21st century; (2) developing a minimum standard of care across the world and; (3) motivating the broad MS community to align standards of care and challenge the current treatment paradigm. The project has been run by an independent agency, iS Health, with the support of an unrestricted educational grant from Merck Serono.

The aim was to develop a consensus statement to reach and influence the broader MS community and serve as a reference tool for stakeholders who can influence the future care of MS patients.

## Methods

### Steering group

Candidates for the MS in the 21st Century Steering Group were identified by iS Health to meet the following criteria: they should comprise key patient groups and leading clinicians active in the treatment and management of MS; and they should represent different groups in different countries worldwide.

The steering group comprises the following members: Peter Rieckmann (Neurologist, Bamberg, Germany); Alexei Boyko (Neurologist, Moscow, Russia); Diego Centonze (Neurologist, Rome, Italy); Alasdair Coles (Neurologist, Cambridge, UK); Irina Elovaara (Neurologist, Tampere, Finland); Eva Havrdová (Neurologist, Prague, Czech Republic); Otto Hommes (Neurologist, European Charcot Foundation, Molenhoek (Nijmegen area), The Netherlands); Jacques LeLorier (Internist Pharmacoepidemiologist and Pharmacoeconomist, Montreal, Canada); Sarah A. Morrow (Neurologist, London, Canada); Celia Oreja-Guevara (Neurologist, Madrid, Spain); Nick Rijke (Interim Director of Policy and Research, UK MS Society, London, UK) and Sven Schippling (Neurologist, Zurich, Switzerland).

### Consensus process

Experts in the area of MS care were asked to be members of the steering committee for the MS in the 21st Century initiative and were invited to participate in a series of three workshop-based steering group meetings between February 2011 and 2012.

Discussions and workshops at the first meeting focused on identifying the most critical goals, the unmet needs, and areas for improvement/development in MS care.

At the second meeting, the group distilled the “areas for development” identified at the first meeting into the highest priority items, which would form the “overarching principles” of the consensus statement. The mechanism for choosing the highest priority items from the initial list of 12 was based on a voting system. Each member of the group was asked to cast six votes to denote their highest priority areas for development. The topics were placed on individual posters and each member was given six red stickers with which to indicate the areas that they considered to present the greatest priority for development. Multiple votes could be cast for a single topic if desired. No firm limit was set as to the number of high-priority areas that would be selected for final inclusion in the consensus statement. Rather, the final items were decided through group discussion.

Once the highest-priority items had been decided upon, the group attempted to define the main points for each principle in relation to the vision “Full access to personalized treatment, with reimbursement, to achieve freedom from disease” in a workshop setting, and to identify what should be done to realize these overarching principles.

The third steering group meeting consisted of a series of discussions aimed at finding resolution on issues raised in the previous meeting and ratifying the draft consensus statement.

## Results

During the first meeting, 12 key areas for development were identified by the group (Table 1).

**Table 1:** Number of votes received for each of the initial 12 “Areas for development” in multiple sclerosis care as identified by the MS in the 21st Century Steering Group

Area for development	Number of votes (%); *n* = 13, six votes per person
Commitment to research	16 (20.5)
Regulatory body education	12 (15.4)
New endpoints in clinical trials	11 (14.1)
Healthcare and social care; personalized care	9 (11.5)
More therapy options	9 (11.5)
MS centers of excellence	7 (9)
Informed, shared decision-making	4 (5.1)
Better communication between stakeholders	3 (3.8)
Cost and reimbursement	3 (3.8)
Drugs with better risk:benefit profiles	2 (2.6)
Early treatment	1 (1.3)
Patient engagement and enablement	1 (1.3)

Each voting group member had a total of six votes. Multiple votes for a single topic were allowed. The group that attended the second meeting and were eligible to vote included eight neurologists, one pharmacoepidemiologist/pharmacoeconomist, three MS group/society members, and one consultant with a long-standing relationship with the MS community in Canada

During the second meeting, the voting process identified which items the group felt were the highest priority topics (Table 1). It was agreed that “early treatment” should be included under the remit of “personalized care”. There was also general agreement that items “patient engagement” and “enabled patients” should be combined, with the revised item termed “patient engagement and enablement”. In addition, a new category, “commitment to research”, was added to the list. The group agreed that seven “overarching principles” should form the basis of the consensus statement: personalized care, patient engagement, commitment to research, regulatory body education and reimbursement issues, new endpoints in clinical trials, more therapy options, and MS centers of excellence.

The main points for each principle in relation to the vision “Full access to personalized treatment, with reimbursement, to achieve freedom from disease” were outlined (Table 2) at the second meeting.

**Table 2:** Feedback on defining the call for action

Areas for development	Acknowledge What are the current challenges?	Develop What needs to be done to achieve the vision for the 21st Century?	Implement How could this be achieved and by whom?	Communicate and engage Who should this be communicated to?
Regulatory body reform^a^	Access to and engagement with regulatory bodies Burdensome regulations driving expense and limiting innovation (Industry, reducing loss) Leading to costly and delayed drugs Different perceptions of risk Opportunity cost Resource cost, i.e., limited pool of patients in trial centers	Improve access to conditional drug approval Promote post-licensing registry Earlier drug approval Learn from diabetes and other conditions Insurance and reimbursement of adverse events	Utilization of a specific opportunity, with coordination of: Patients/patient societies Neurologists Industry	EU Patients Neurology advisory groups Key opinion leaders Agencies
Commitment to research	Reduced charity income Reduced government funding for basic research	A culture of research embedded in all aspects of MS	MS centers of excellence Research networks Leverage of funds	Government departments for health and research Foundations EU
New endpoints in clinical trials (and new designs of trials)	Optical coherence tomography, MRI techniques Subgroup analyses No consensus currently on outcome measures and clinical trial design Neuroprotection Fatigue measurement and recognition Composite endpoints and scales New types of clinical trials	Achieve consensus from expert groups with endorsement of regulatory authorities Development of new composite endpoints/scales “De-risking” clinical trials	Physician (expert neurologist)/patient/industry Regulatory bodies Experts	Regulatory Industry Funders of clinical research Academic Charities Experts
Personalized care (healthcare and social care)	Fragmentation of care into different departments Ability to effect change due to governmental structures and processes	Integration and coordination Provision of information—‘navigating the system’ Patient adherence Effective partnership	Empowerment of MS nurses as central coordinators MS centers of excellence Patient engagement Shared decision-making Education for patients and physicians	Coordination and engagement of other patient organizations Coalition of stakeholders Politicians and bureaucrats
More therapy options	Cost/affordability Safety Tolerance High level of regulatory demands Relevance of placebo arm? Long-term data on therapies Standard approach to all kinds of treatment	Decrease cost and increase available resources Cost-effectiveness strategies Widen focus of research Education and advocacy Public support for longitudinal patient registries	Use expertise from other fields Patient advocacy Make additional resources available for unmet needs Incentivize investments following specific public health interests Widespread educational programs	Regulatory bodies and funding agencies Political decision-makers General public Governments Neurological societies
MS centers of excellence	Quality variation Regional differences Different resources Different levels of experience in clinical trials	Multidisciplinary approach developed Define main standards (country-specific) Full access to information Systems and educational programs	Strengthen current networks Export and share positive experiences Open window for more centers to participate in clinical trials and research	Healthcare authorities Current networks World Health Organization (WHO) European Commission European and country parliaments

*EU* European Union, *MS* multiple sclerosis

^a^Wording changed from “education” to “reform” in the breakout session

The consensus statement below represents the culmination of discussions at the three steering group meetings and aims to present a strong vision for what the treatment and care of MS should look like in the future.

### Consensus statement

To realize our vision of full access to personalized treatment, with reimbursement, to achieve freedom from disease, we call upon healthcare providers and decision-makers to address simultaneously the following overarching principles that will guide patient management, in order to improve outcomes for people with MS.

#### Personalized care

We need personalized treatments for all types of MS [10, 11]. This means developing new approaches that incorporate a wide range of pharmacological and non-pharmacological strategies that focus on the needs of people living with the disease as individuals, and aim to reduce disease activity, slow disability progression and improve management of symptoms such as depression, immobility, fatigue, and others, which can be caused by MS [10–12].

MS care requires diverse therapies and strategies to address the complexity of the disease’s considerable symptoms and challenges [13]. Governments, healthcare decision-makers, and healthcare systems must be prepared to see MS patients as requiring interventions beyond greater access to disease-modifying agents alone, especially for those with progressive forms of MS.

The best treatment regimen is often specific to the patient and best achieved through continuity of care with a team of physicians and allied professionals [11, 12]. Healthcare decision-makers must realize that this outcome is often compromised in modern hospitals, where, in the name of efficiency, patients will generally see a sequence of different doctors and may receive increasingly fragmented care. To overcome this barrier, we call on healthcare policy-makers to prioritize the development of centers of excellence for improved access to personalized treatment and to empower MS nurses as central coordinators for patient care.

Patients also have the right to be informed about, and involved in, all decisions regarding their treatment, including early therapy options [11]. All available therapies and their respective risks and benefits should be communicated to patients by their physicians, and patients should also have access to other reliable, independent sources to ensure that they are empowered to make informed decisions [11, 14–16]. In addition, affording patients psychological and social support as part of their treatment package is likely to ensure the greatest possible mitigation of the potential financial, social, and psychosocial burdens associated with MS [8, 12, 17–22].

#### Patient engagement

Patients should be engaged as advocates. They should be empowered to drive negotiations with regulators and payers (see example below of how this has been achieved in the field of HIV). In addition, patient organizations should work more closely with clinicians, industry, and regulatory bodies to petition for and secure research funds.

An example: How patient engagement has helped improve outcomes for people with HIV

- Improvements in HIV treatment began when well-organized patient groups started collaborations with other stakeholders. This was during a period of significant political and societal indifference.
- In 1983, the advisory committee of the People with AIDS developed the “Denver Principles”, which empowered people with AIDS/HIV by changing how they were viewed and treated.
- The organization ACT UP (the AIDS Coalition to Unleash Power) who were committed to direct action to end the AIDS crisis, made demands including better access to drugs as well as cheaper prices, public education about AIDS, and the prohibition of AIDS-related discrimination.
- Other ways in which patient organizations have played a role in improving outcomes in HIV:
  - Treatment information and disease management
  - Involvement in drug development and clinical trials
  - Increased institutional involvement in regulatory agencies
  - Scientific societies
  - Discussions with payers and Health Technology Assessment (HTA) agencies

Learnings for MS care from the HIV initiative

- There are a similar number of patients with HIV and MS in Europe (approximately 600,000–700,000). However, the awareness of MS in society is well below that of HIV.
- A similar program to that adopted by the HIV community, which motivates the broad MS community to align behind a call to action and strongly challenges the current treatment paradigm, would be beneficial.

#### Commitment to research

We need to embed a culture of research in all aspects of MS care. The continued progress of basic research is currently under threat due to reduced charity income and governmental funding. However, research across all domains is key for a better understanding of disease mechanisms, which ultimately leads to more effective new treatment options (pharmacological and non-pharmacological), including therapies for progressive forms of MS.

We recommend the development of an international network of MS centers of excellence (see below the MS centers of excellence) as a platform for establishing research networks and leveraging funding.

In addition, without compromising patient safety, we call upon regulators to re-evaluate the existing European Commission (EC) requirement for investigator-led hypothesis-generating studies to meet the same standards as industry-funded clinical trials, as this criterion has severely limited the scope of such investigator-led studies.

#### Regulatory body education and reimbursement issues

The authors believe that patient groups, clinicians, and industry need to have greater access to regulatory bodies at a national and local level in order to:

- Improve the regulator’s understanding of the complexities associated with the treatment and care of MS patients
- Align perceptions of the risks of the disease and its treatment.

The introduction of a conditional license, allowing several thousand patients to be treated for a number of years, would allow safety data to be formally gathered from people with MS who are willing to take greater risks and be exposed to greater uncertainty.

Regulatory bodies need to consider how escalating regulatory requirements, including increasing demands for long-term safety data, could be major drivers for spiraling development costs [23–25]. This leads to increasing drug prices, limited innovation, and delays in getting promising drugs to patients.

Reimbursement agencies should consider the substantial indirect costs associated with MS [3, 26–37] and the related burden to society in their calculation of cost:benefit ratios.

#### New endpoints in clinical trials

Clinical trials in MS are still largely based on single-parameter measures of disease relapses or disability progression [38]. Other parameters, such as subclinical inflammatory activity, neuroprotection, lesion burden detected by MRI, health economics, cognitive impairment, and fatigue are regarded only as secondary endpoints or are rarely measured at all. Employing the use of single-parameter measures in clinical trials can fail to measure the effect of treatment on the actual disease process, and these strategies only partly reflect real-world treatment scenarios. Developing better patient- and physician-reported tools are needed for the assessment of these parameters, and using those within composite endpoints may, therefore, improve measures of treatment efficacy. In fact, a number of groups have recently used composite endpoints in phase 3 clinical trials with success [39–44].

New clinical trial designs and endpoints that enable effective treatment development and evaluation to achieve relevant benefits are needed [38]. Such endpoints should include patient-reported measures, clinical and functional assessments, and biomarkers [45–47]. We encourage the development of initiatives involving regulatory authorities, expert groups, and industry, which will amalgamate these disparate elements into new composite endpoints/scales [45].

#### More therapy options

Although there are already multiple pharmacological treatment options available for MS, the high costs of these treatments may limit patient access [48]. Governments, regulators, funding agencies, clinicians, the pharmaceutical industry, and patient groups need to work together to develop strategies to deliver more cost-effective medicines. They also need to widen the focus of research to ensure the continuous development of better therapy options with improved efficacy, safety, and tolerability profiles.

Patients should have greater access to comprehensive care regimens that include symptomatic care, rehabilitation, and psychological support alongside pharmacological solutions [11].

Non-responding patients and those with aggressive disease should be allowed access to experimental treatment options in well-controlled programs. We petition regulatory bodies and funders to provide this access. In addition, we ask regulators and funders to consider the relevance of the placebo arm and the assimilation of more long-term data on the therapy options available. In this regard, we support the European Commission’s consideration for a patient registry [4].

#### MS centers of excellence

We need a network of dedicated MS centers of excellence that meet a set of predetermined minimum standards and demonstrate a will to contribute to research and to share resources. This is to improve diagnosis and treatment, as well as to provide optimum patient support [12].

### Potential benefits of the consensus statement on future MS care

This statement is intended to raise awareness of future MS care and has the overall aim of affording MS patients freedom from disease. Initiatives that disseminate information about potential gaps in care identified by MS experts may give rise to adoption of practices that promote improved future care of MS. Should the elements of this consensus statement be integrated into MS care, we foresee the following benefits:

- Ensure patients fully understand their treatment options
- Ensure healthcare decision-makers understand the complexities of MS and the degree to which patients are willing to accept risks of therapies
- Overcome the stigma associated with MS to achieve better social integration of people with MS
- Develop strategies to improve the cost-effectiveness of MS medicines
- Ensure patients have access to more treatment options
- Improve communication between clinicians, regulatory authorities, and healthcare bodies.

## Discussion

The steering group of the “MS in the 21st Century” initiative believes that it is the right of every patient to have access to early diagnosis, more and better treatment options, rehabilitation and regeneration strategies, and effective management of MS symptoms.

This publication is a call to all regulators, healthcare providers and decision-makers, clinicians, industry representatives, and patient groups within the MS community to work together to ensure that all people affected by MS have full access to personalized treatment, with reimbursement. Our vision is for people affected by MS to have freedom from disease.

The steering group acknowledges that for this initiative to be successful it will be imperative to engage appropriate stakeholders, including regulators and patients, and develop a strong collaboration with them to ensure that the principles underlying the statement are accomplished. Members of the steering group are currently seeking endorsement of the consensus statement from individuals, as well as professional and patient organizations. The highest priority groups with which to engage were identified as specific agencies, national MS and neurological societies, academic groups, and patient organizations.

## Limitations

One limitation of this consensus statement is that it is based primarily on the opinions of experts who agreed to participate in the MS in the 21st Century initiative. As such, it will naturally be skewed to the personal opinions of those attending (a large proportion of who were neurologists). However, the group has attempted to overcome this, in part, by further disseminating the consensus statement to other bodies, requesting their endorsement and further comments.
